# Vasovagal Syncope at Work: A Narrative Review for an Occupational Management Proposal

**DOI:** 10.3390/ijerph20085460

**Published:** 2023-04-11

**Authors:** Anna Rita Corvino, Vincenzo Russo, Maria Grazia Lourdes Monaco, Elpidio Maria Garzillo, Daniele Guida, Angelo Comune, Erika Parente, Monica Lamberti, Nadia Miraglia

**Affiliations:** 1 Experimental Medicine Department-Hygiene, Occupational, and Forensic Medicine Division-Occupational Forensic Area, University of Campania “Luigi Vanvitelli”, 80138 Naples, Italy; annarita.corvino@unicampania.it (A.R.C.); daniele.guida1@studenti.unicampania.it (D.G.); monica.lamberti@unicampania.it (M.L.); nadia.miraglia@unicampania.it (N.M.); 2 Department of Medical Translational Sciences, University of Campania “Luigi Vanvitelli”—Monaldi Hospital, 80131 Naples, Italy; vincenzo.russo@unicampania.it (V.R.); anzcomune@gmail.com (A.C.); parente-erika@libero.it (E.P.); 3 Occupational Medicine Unit, University Hospital of Verona, 37134 Verona, Italy; 4 Department of Prevention, Abruzzo Local Health Unit No. 1, 67100 L’Aquila, Italy

**Keywords:** vasovagal syncope, worker management, occupational safety risk, return to work

## Abstract

Syncope is a complex clinical manifestation that presents considerable diagnostic difficulties and, consequently, numerous critical issues regarding fitness for work, especially for high-risk tasks. To date, it is impossible to quantify the exact impact of syncope on work and public safety since it is highly improbable to identify loss of consciousness as the fundamental cause of work or driving-related accidents, especially fatal injuries. Working at high-risk jobs such as public transport operators, in high elevations, or with exposure to moving parts, construction equipment, fireworks, or explosives demand attention and total awareness. Currently, no validated criteria or indicators are available for occupational risk stratification of a patient with reflex syncope to return to work. By drawing inspiration from the updated literature, this narrative review intends to summarise the leading knowledge required regarding the return to work for subjects affected by syncope. According to the available data, the authors highlighted some key findings, summarised in macro-items, such as defined risk stratification for vasovagal accidents, return to work after a critical event, and a focus on pacemaker (PM) implementation. Lastly, the authors proposed a flowchart for occupational physicians to help them manage the cases of workers affected by syncope and exposed to levels of risk that could represent a danger to the workers’ health.

## 1. Introduction

Transient loss of consciousness (T-LOC) from cerebral hypoperfusion is known as syncope. It is characterized by a sudden onset of temporary loss of consciousness and complete spontaneous recovery. It is often preceded by some symptoms (dizziness, nausea, weakness, sweating, and visual disturbances) that is a prelude to its arrival. Syncope shares many clinical features with other T-LOC; it, therefore, presents in many differential diagnoses. The latest classification of syncope is based on its leading cause, emphasizing groups of disorders with common pathophysiology, presentation, and risk; it includes (1) reflex (neuro-mediated) syncope (vasovagal, situational, and carotid sinus syndrome); (2) syncope due to orthostatic hypotension (OH); (3) cardiac syncope due to arrhythmias or structural heart disease [1]. Other causes of non-syncopal T-LOC include epilepsy, psychogenic forms, endocrinological disorders (diabetes insipidus resulting in volume depletion), hypoglycemia, hypoxia, and hyperventilation with hypocapnia [2].

The most common syncope at all ages and settings is reflex syncope. The second most frequent reason is cardiac syncope. Studies show a large variation in the proportion of individuals with a cardiac cause; higher frequencies are noted in emergency situations, mainly in elderly patients and in cardiology-focused environments. Children, teenagers, and young adults rarely have cardiac syncope. OH is a rare cause of syncope in individuals under the age of 40, whereas it is common in people beyond the age of 40 [3].

In addition to clinical risk stratification, it is necessary to consider all aspects that could be influenced by the onset of syncope (whether it has a rapid or slow onset) and its potential recurrence during the patient’s daily life, such as during their work and the probable relationship of syncope with it. To date, it is impossible to quantify the exact impact of syncope on work and public safety since it is highly improbable to identify loss of consciousness as the fundamental cause of work or driving-related accidents, especially fatal injuries [4,5].

The damage to the worker or third parties could be substantial if the T-LOC occurs during a high-safety-risk work activity. Working at high-risk jobs such as public transport operators, high altitude jobs, or jobs with exposure to moving parts, construction equipment, fireworks, or explosives demand attention and total awareness. Currently, there is a lack of literature regarding managing workers suffering from reflex syncope in work-related contexts other than driving but still at high risk for safety.

There are currently no validated criteria or indicators available for the stratification of occupational risks of a patient with reflex syncope who must resume his work activity [3]. Within this context, this narrative review aimed to screen the scientific literature to provide evidence-based suggestions and preventive measures in managing workers suffering from reflex syncope, particularly from the occupational physician’s point of view.

The literature search was limited to published papers on one database (PubMed) in English over the past ten years. Epidemiologic studies about syncope incidence and prevalence in general and working populations were also included to give an idea of the topic’s relevance in occupational contexts. No quality assessment was performed, and results are presented as a narrative summary. 

## 2. Syncope Occurrence

As reported by the International Labor Organization in a report ranging between 2000–2016, occupational injuries caused 360,000 deaths and represented the risk factor that contributed to the greatest loss of DALYs globally in 2016 as well [6]. According to the EUROSTAT Health and Safety at Work in Europe [7], loss of control, slipping, stumbling, and falling are the three main causes of workplace accidents. It is feasible to hypothesize that an occult syncope or a pre-syncope leading to a loss of control might also be a factor in this environment, where a lack of work safety procedures is likely to be a common reason accounting for these mishaps [4].

The incidence of syncope is particularly high in early working age [8,9]. Nevertheless, little data on its effects on workplace safety is available. Given that one-third of patients have recurring syncope within a few years, and that syncope has a lifetime prevalence of 35% [10,11], it is important to take workplace syncope into account. Compared to the general population, patients with a history of syncope had a nearly twofold increased risk of motor vehicle accidents, according to a recent study [12]. However, it is unknown if syncope may be linked to occupational accidents [4,5]. 

Several studies have demonstrated that, in addition to the immediate effects of syncope, syncope is linked to a lowered quality of life, such as patients with chronic illnesses [13,14]. It is conceivable that interference with employment and work life is one of the primary causes of the poor quality of life experienced by syncope patients. In research, half of the syncope patients said that it affected their ability to work [13].

A Danish retrospective cohort study found that syncope was linked to negative impacts on employment and occupational safety: the probability of occupational accidents was 1.4 times higher in syncope patients than in the general population, and one out of three lost their jobs within two years [15].

The above-reported data show that the impact of syncope on employment deserves further research, especially examining in detail the prevalence of syncope in the workplace to facilitate a better understanding of the content and organization of preventive strategies.

## 3. Patient-Worker-Management 

### 3.1. Diagnosis and Treatment 

The preliminary step in the diagnostic evaluation of suspected syncopal TLOC consists of an accurate history of present and previous attacks, physical examination, supine and orthostatic blood pressure measurements, and electrocardiogram (ECG). The Calgary Syncope Symptoms Score can be an efficient diagnostic tool to identify patients with vasovagal syncope with 89% sensitivity and 91% specificity, combining seven clinical features of anamnestic and electrocardiographic nature [16]. The CSSS has been validated in the younger population with no evidence of structural heart disease; however, it shows a lower degree of sensitivity and specificity in the elderly population, primarily in the presence of mild cardiovascular disease and increased prevalence of cardiovascular risk [17].

Additional examinations may be performed when needed (1) fast ECG monitoring in cases of suspected arrhythmic syncope; (2) echocardiography in cases of previously diagnosed heart illness, evidence of structural heart disease, or syncope secondary to a cardiovascular etiology; (3) carotid sinus massage (CSM) in patients older than 40; (4) blood tests such as troponin when cardiac ischemia-related syncope is suspected, hematocrit or hemoglobin when bleeding is suspected, oxygen saturation and blood gas analysis when hypoxia is suspected, or D-dimer when pulmonary embolism is detected). After a preliminary clinical evaluation, the head-up tilt test (HUTT) is a useful and effective diagnostic technique for individuals with suspected reflex syncope [18]. Moreover, it can be used to evaluate the cardioinhibitory response with asystole as the main determinant of syncope (Table 1) [19,20,21]. 

The main goals of the treatment of neuro-mediated syncope are the prevention of syncopal recurrences and associated trauma and the improvement of the quality of life. The cornerstone of management is based on patients’ education, lifestyle modification, and reassurance regarding the benign nature of the condition. Avoiding triggers (e.g., hot and crowded environments, prolonged orthostatic position, hypovolemia, coughing, a too-tight collar, etc.) and increased oral hydration and salt intake is further crucial. Similarly, the discontinuation/reduction in hypotensive therapy targeting a systolic BP lower than 140 mmHg, support stockings, physiologic counterpressure techniques, and tilt training can significantly reduce syncope recurrence. 

Recurrent and unpredictable reflex syncope, even if it has a benign course, can be disabling and may require patients to undergo pharmacological treatment, especially those that present with recurrent syncope with a very short prodrome phase or without it, as it can expose the patient to the risk of trauma especially when syncope occurs during high-risk activities (e.g., operating machinery, driving, or flying, etc.). The use of fludrocortisone (0.05–0.2 mg once daily) or midodrine (2.5–10 mg, three times daily) may be effective in reducing recurrent syncope in young patients with low-normal BP values and no comorbidities.

The evidence about the optimal pharmacological therapies may be troubled by methodological issues such as the paucity of randomized controlled studies, the small size of study cohorts, and the short follow-up duration.

Dual chamber pacing is recommended in extremely selective reflex syncope patients who are older than 40 and have severe, repeated, unpredictable syncopal episodes with confirmed asystole caused by either CSM, tilt testing, or monitored using a monitoring system [22,23]. The design of closed-loop stimulation, one of the pacing algorithms that are now available, is expected to trigger pacing early and at a sufficient rate before a drop in blood pressure, as has been seen during the tilt test [24,25,26,27]. 

A well-structured clinical-diagnostic procedure is a benchmark not only for the clinical management of the patient but also plays a fundamental role, with social and economic repercussions, on the patient’s quality of life and work.

### 3.2. Risk Stratification

To improve a diagnostic and therapeutic approach, risk classification of patients who present to emergency departments (ED) with syncope is crucial. Several scales are available in the literature to help the emergency physician stratify the clinical risks of patients with syncope to either guide them to hospital admission or discharge them after appropriate outpatient assessment [28,29,30]. However, after the stratification of the clinical risks, it is also necessary to consider all other aspects that could be influenced by the occurrence of syncope and its potential recurrence during the patient’s daily life, such as the patient’s job and if there is an association of syncope with it. Surely, the resumption of a dangerous job must be subordinated to a good diagnostic and therapeutic process and to a stratification of the risk of accident and must certainly include the necessary precautions in order to avoid endangering the life of the patient and the possible third parties. In addition, there is a lack of individualized advice regarding the timing and safety of driving and work resumption after syncope. This could be the cause of an unjustified delay in returning to work and could lead to a negative psychological impact on the patient. However, it could be harmful if the patient decides on his/her own to resume a high-risk job without proper precautions. This could lead to higher costs for society and employers.

No validated criteria or indicators are currently available for the stratification of the occupational risk of a patient with syncope who must resume his work activity. 

Only the Canadian Cardiovascular Society has provided some numerical indicators which, starting from a risk of road accident due to syncope considered acceptable (1/20000 per year), allow to establish which patients can resume driving and how long after a syncopal event. These indicators depend on multiple factors such as driving time, the type of vehicle, the risk of recurrence at 1 year, and the likelihood that an accident will actually occur as a result of syncope while driving [31].

Work risk stratification has a primary role in identifying and managing all those workers exposed to tasks with a high intrinsic risk.

### 3.3. Return to Work

Several studies have suggested that syncope is associated with a reduced quality of life comparable to that of patients with chronic disorders [13,14]. Probably, one of the main reasons for the poor quality of life after syncope could be the relationship with work activity. Van Dijk et al. reported that half of the patients with syncope described that it influenced their ability to work [13]. However, there is a significant lack of literature on the subject as a large sector of the working population is not involved.

The first study to show a substantial correlation between syncope and safe work performance, including occupational injuries and job termination, involved individuals with syncope in Denmark [8]. The study’s key findings showed that occupational accidents in syncope patients were more common among employed people with syncope than among the general employed population and that risk rose by an additional factor of 1.4 among those who experienced recurrent syncope [9]. The risk of any outcome was highest in patients with younger age, comorbidities, or unfavorable socioeconomic status; 91.6 percent of people with syncope maintained or returned to work after hospitalization [10]; however, the 2-year risk of termination of employment after syncope was 31.3 percent, which was two folds of the reference population [11].

In the study by Sakaguchi and Li, the authors reviewed different guidelines for resuming private driving for patients with vasovagal syncope. The main finding of the review was that patients with a low risk of syncope recurrence, i.e., less than 22%, can return to driving with minimal or no restrictions [32]. Sorajja et al. discussed the possible role of driving itself as a potential cause of syncope [33]. Out of 3877 patients who experienced syncope during the study period, 381 workers (9.8%) had syncopal episodes while driving. In 37.3% of cases, the main cause of syncope while driving was identified as neurally mediated, which is uncommon in a seated position, while cardiac arrhythmias represented only 11.8% of the causes. The authors suggested several mechanisms to explain the higher frequency of neuro mediated syncope among drivers with syncope, such as unfavorable microclimate and exceedingly high temperatures inside the vehicle, strong emotional stimulation while driving, and venous-type alterations [34]. It should be noted that the risk of syncope recurrence and the causes of syncope were both similar in patients who fainted while driving and those with non-driving related syncope. Therefore, the clinical management of workers who experience syncope while driving may be similar to those in the general syncope patient population [10,35,36].

Given the lack of convincing data about the relationship between syncope and various work activities, to date, only recommendations regarding professional driving have been established, while there are no suggestions regarding the execution of professional activities with high accident risks [37,38,39,40]. It should be stressed that several characteristics that can cause neurally induced syncope may be present in some typical working activities [41]. Indeed, high ambient temperatures, high humidity, and rapid temperature variations are examples of an unfavorable microenvironment, besides wearing heavy protective clothing, which may be a contributing factor to temporary orthostatic intolerance or presyncope, which in turn causes syncope. In this scenario, it is crucial to point out that certain low-cost adjustments in the physical setting of the workplace, gained, for example, by placing an air conditioner, which might remedy the worker’s problem.

The results of a survey carried out in preparation for the First International Workshop on Risk Stratification of Syncope in the Emergency Department reported that 96% of 27 expert physicians consider the patient’s work history essential in managing Emergency cases of syncope. Among the doctors interviewed, 88% reported that, in the face of a lack of evidence-based recommendations in the literature, the management of patients, particularly their return to work, was mainly based on individual experience [5].

Barbic F et al. conducted a study to assess a newly developed methodology for occupational task risk assessment in syncope patients. The 149 occupational physicians (OP) with ten years of clinical experience were asked to complete a Visual Analogue Scale during a workshop on syncope and occupational risk, taking into account the potential harm to the worker and the likelihood of accidents occurring if syncope occurred during the job task. Five job tasks were identified, characterized by different safety risks (1, driving; 2, toxic products handling; 3, job performed closed to hot surfaces or free flames; 4, surgical activity; 5, office job). In patients with syncope, OP correctly classified the risk posed by the various work responsibilities. Unexpectedly, task #3 was assigned a risk level comparable to that seen in drivers., highlighting that even working activities other than driving should be considered at risk. These data are of extreme clinical and social importance in the occupational management of patients with syncope, especially when they are expected to return to perform tasks with a high risk of injury to themselves or third parties [3].

According to Barbic F. et al., an individualized approach should be achieved, considering the risk of syncope recurrence and the severity of syncope while performing a risky job, such as driving [5]. Cooperation between the cardiologist, an expert in syncope, and the occupational physicians could provide global management of patients by improving their quality of life, avoiding the formulation of hasty judgments of unfitness for work and, therefore, unjustified removal from work with a large cost cutting for the community. Based on stratifying the risk of syncope patients associated with high-risk jobs for safety, the proposed model needs validation by controlled and ad hoc studies. These studies should first assess the accuracy of the proposed model in patient risk stratification after syncope based on a specific job.

**Figure 1:**
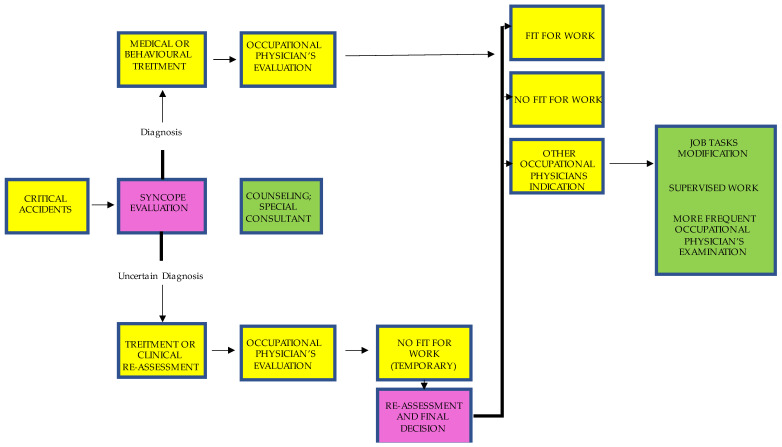
Fitness for work of employees affected by syncope and engaged in high-risk job tasks.

Return to work must be assessed by an experienced occupational physician, and changes to the work environment or organization may be suggested. A period of removal from high-risk job tasks (3 to 6 months or more) and regular follow-up are essential for monitoring clinical fitness to return to work (see Figure 1). A multidisciplinary approach, including continuous training on occupational safety, could reduce occupational injuries due to syncope relapses and promote a safe and appropriate return to work for patients with syncope.

### 3.4. Focus on Fitness for Work after Pacemaker (PM) Implantation

There are few studies in the literature investigating workers’ return to work suffering from vasovagal syncope treated with a *PM* implant. Workers affected by cardioinhibitory syncope treated with a *PM* implant could have problems returning to work, especially in the case of work environments with exposure to electromagnetic radiation. One of the most frequent exposures to electromagnetic radiation in the workplace is due to spot, industrial welding machines, and electric motors, which could represent a problem for exposed workers who have recently undergone PM implantation. While welding equipment in the 100–200 amperes range is unlikely to produce significant electromagnetic interference (EMI), welding with industrial power equipment more significant than 500 amperes can be a problem as it can inhibit pacemakers.

Therefore, for any pacemaker-dependent patient, it is paramount to assess potential EM sources in the work environment before the patient is allowed to return to their occupation to ensure that the pacemaker is not inhibited [40].

Currently, there are no clear regulations regarding the safe management of pacemakers or ICD workers exposed to high electromagnetic fields (EMF). The interim European directive on electromagnetic fields (2004/40/EC) requires employers to consider the safety of workers at particular risk. In workplaces with high levels of EMF exposure, employees with PMs or ICDs are generally removed from the workplace and are assigned to other tasks or are paid a pension with additional costs to employers and society, in addition to the negative psychological implications for the worker. 

In pacemakers or their interrogation telemetry, static magnetic fields (0 Hz) created by objects with permanent magnets have been demonstrated to cause EMI. Exposure to a static magnetic field in pacemakers can trigger a change to an asynchronous pacing mode. Examples of equipment that can create static magnetic fields include hard drives in portable media players and laptop computers, as well as small neodymium magnets and wireless headphones. Similarly, magnetic resonance imaging equipment’s static magnetic fields frequently create EMI. However, the data on EMI from alternating extremely low-frequency magnetic fields (<50 Hz) are scanty. 

Tiikkaja M. et al. [42], in a study to evaluate the susceptibility of pacemakers to external electromagnetic fields, found no interference with bipolar pacemakers, even from reasonably high magnetic fields. Employees can usually return to work following a bipolar pacemaker implant based on an appropriate risk assessment. However, it was pointed out that unipolar pacemakers had much interference in identical fields. One of the pacemakers was also hampered by the EAS gate and welding cable. These findings validate the current practice of including bipolar settings in new pacemakers. When possible, avoid using unipolar configurations since they can put pacemaker users in life-threatening circumstances in EMF surroundings.

According to Taino et al., it is necessary to apply specific risk assessment criteria to correctly manage PM or ICD carriers’ return to work, considering the lack of specific procedures and indications for the occupational medicine specialist [43]. The information gathered and the literature data suggest that welding can represent a risk for a subject with a PM implant. Given the absence of precise protocols and guidelines for the occupational medicine expert, Taino et al. contend that particular risk assessment criteria must be used to manage PM or ICD carriers’ return to work [43].

We think this study could support workplace prevention and health promotion initiatives.

## 4. Conclusions

This review shows a lack of literature regarding the management of workers suffering from reflex syncope in work contexts other than driving but still at high risk for safety. There is no shared protocol for managing a worker with syncope and the patient’s return to work after an episode of syncope. An individualized approach should be achieved, taking into account the risk of syncope recurrence and the severity of syncope while performing a risky job. 

Cooperation between the cardiologist, an expert in syncope, and the occupational physicians could provide global management of patients by improving their quality of life, avoiding the formulation of hasty judgments of unfitness for work and, therefore, unjustified removal from work with a considerable cost cutting for the community. A period of removal from high-risk job tasks (3 to 6 months or more) and regular follow-up are essential for monitoring clinical fitness to return to work. A multidisciplinary approach, including continuous training on occupational safety, could reduce occupational injuries due to syncope relapses and promote a safe and appropriate return to work for patients with syncope.

On the other hand, the lack of literature also concerns the management of a worker suffering from vasovagal syncope treated with a PM implant. Moreover, in this case, an individualized and multidisciplinary approach is required, considering the type of occupational exposure to high-frequency electromagnetic fields. Therefore, for any pacemaker-dependent patient, it is paramount to assess potential EM sources in the work environment before the patient can return to their occupation to ensure that the pacemaker is not inhibited.

## Figures and Tables

**Table 1:** Classification of head-up tilt test-induced vasovagal syncope.

Response	Definition
Mixed	HR decreases >10% with respect to maximum value during the test, minimal HR> 40 bpm or less than 40 bpm for less than 10 s. BP falls before HR.
Cardioinhibitory without asystole	HR decreases with minimal HR < 40 bpm for more than 10 s and no asystole for more than 3 s. BP falls before HR.
Cardioinhibitory with asystole	HR decreases with asystole for more than 3 s. HR drop coincides with or precedes BP fall.
Vasodepressive	BP decreases. HR not decreases or <10% of the maximal value during the test.

HR: heart rate; BP: blood pressure; bpm: beats per minute.

## Data Availability

The data presented in this study are available on request from the last author.
